# Lymphatic System and the Kidney: From Lymphangiogenesis to Renal Inflammation and Fibrosis Development

**DOI:** 10.3390/ijms25052853

**Published:** 2024-03-01

**Authors:** Elodie Stasi, Savino Sciascia, Carla Naretto, Simone Baldovino, Dario Roccatello

**Affiliations:** University Center of Excellence on Nephrologic, Rheumatologic and Rare Diseases (ERK-Net, ERN-Reconnect and RITA-ERN Member) with Nephrology and Dialysis Unit and Center of Immuno-Rheumatology and Rare Diseases (CMID), Coordinating Center of the Interregional Network for Rare Diseases of Piedmont and Aosta Valley, ASL Città di Torino and Department of Clinical and Biological Sciences, University of Turin, 10154 Turin, Italysimone.baldovino@unito.it (S.B.)

**Keywords:** lymphatic system, kidney, lymphangiogenesis, renal fibrosis

## Abstract

The lymphatic kidney system plays a crucial role in managing interstitial fluid removal, regulating fluid balance, and tuning immune response. It also assists in the reabsorption of proteins, electrolytes, cytokines, growth factors, and immune cells. Pathological conditions, including tissue damage, excessive interstitial fluid, high blood glucose levels, and inflammation, can initiate lymphangiogenesis—the formation of new lymphatic vessels. This process is associated with various kidney diseases, including polycystic kidney disease, hypertension, ultrafiltration challenges, and complications post-organ transplantation. Although lymphangiogenesis has beneficial effects in removing excess fluid and immune cells, it may also contribute to inflammation and fibrosis within the kidneys. In this review, we aim to discuss the biology of the lymphatic system, from its development and function to its response to disease stimuli, with an emphasis on renal pathophysiology. Furthermore, we explore how innovative treatments targeting the lymphatic system could potentially enhance the management of kidney diseases.

## 1. Introduction

The mammalian body hosts two primary circulatory networks: the blood vascular system and the lymphatic vascular system.

The lymphatic system plays a crucial role in maintaining bodily equilibrium by removing leaked interstitial fluid and supporting the movement and activation of immune cells. Within the kidney, lymphatic vessels are primarily located in the cortex, while in the medulla, the ascending vasa recta function as lymphatic-like vessels, aiding in the reabsorption of interstitial fluid. While most of the lymphatic network originates from the venous system, there is evidence indicating the presence of lymphatic structures arising from nonvenous sources. After their initial formation, the number of lymphatic vessels tends to stay consistent, although they can adapt dynamically to the needs of the surrounding tissue.

Pathological situations, including tissue damage, excess interstitial fluid, high blood sugar levels, and inflammation, can trigger lymphangiogenesis—the formation of new lymphatic vessels. This process is linked to several kidney diseases, such as polycystic kidney disease, hypertension, challenges with ultrafiltration, and issues following organ transplantation. While lymphangiogenesis serves beneficial roles in removing excess fluid and immune cells, it can also contribute to a cycle of inflammation and fibrosis within the kidneys.

In this review, we aimed to discuss the lymphatic system biology, from its origins and functionality to its reaction to disease triggers with a specific focus on renal pathophysiology. Additionally, we explored how novel treatments specifically targeting the lymphatic system could potentially improve the management of kidney diseases. 

## 2. Lymphatic Development

**Figure 1:**
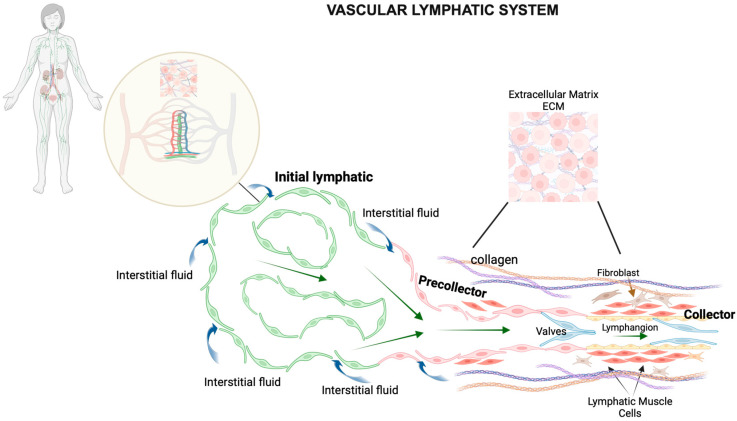
Schematic representation of the vascular lymphatic system. The lymphatic system comprises a widespread network of vessels that extend to nearly all vascularized tissues in the body, often running alongside blood vessels and playing a crucial role in fluid balance. This system, together with various lymphoid tissues and organs, such as lymph nodes distributed throughout the body, orchestrates the regulation of immune cell functions. Within the small intestine, Peyer’s patches—specialized lymphoid follicles—operate similarly to lymph nodes by overseeing immune surveillance and coordinating immune responses in the intestinal lining. Lymphatic capillaries feature unique, oak leaf-shaped endothelial cells with overlapping edges, forming discontinuous, button-like junctions. These specialized junctions allow the capillaries to efficiently absorb interstitial fluid from the surrounding tissue. In contrast, the endothelial cells of collecting lymphatic vessels are connected by continuous, zipper-like junctions, which make them less permeable. Both types of lymphatic endothelial cells (LECs)—those in capillary and collecting vessels—are bordered by similar proteins forming adherens and tight junctions, ensuring their structural integrity. The structure of lymphatic capillaries is distinctively designed for optimal function; they possess a sparse, discontinuous basement membrane and do not have mural cells (smooth muscle cells and pericytes that provide structural support), which facilitates their ability to absorb interstitial fluid, various molecules, and immune cells efficiently. These capillaries then lead into a hierarchical system of collecting lymphatics. The collecting vessels are characterized by the presence of smooth muscle cells (SMCs) in their walls and internal valves that ensure lymph flows in one direction, preventing backflow and ensuring efficient lymph transport and drainage.

Lymphatic vessels originate from the differentiation of embryonic venous blood endothelial cells (BECs) situated along the anterior side of the cardinal vein [1,2,3,4]. Table 1 summarizes the main genetic, molecular, and cellular pathways involved in developing lymphatic vessels. This process commences with the activation of the transcription factor Prospero homeobox 1 (PROX1) in venous blood endothelial cells (BECs), triggered by the actions of the sex-determining region Y box 18 (SOX18) and the chicken ovalbumin upstream promoter transcription factors (COUP-TFII). The specification of lymphatic endothelial destiny is governed by Prox1, evident from the absence of lymphatics and subsequent failure to thrive in Prox1-knockout mice [1,5]. These lymphatic endothelial cells (LECs), originating from the cardinal vein, express α9 integrin and VEGFR3, facilitating their migration towards mesenchymal cells guided by Vascular Endothelial Growth Factor C (VEGFC) gradients. The appearance and migration of lymphatic endothelial cells (LECs) necessitate the presence of VEGFC, whose activity is regulated by proteolytic cleavage facilitated by two proteins: collagen- and calcium-binding EGF domains 1 (CCBE1) and a metalloproteinase known as a disintegrin and metalloproteinase with thrombospondin motifs 3 (ADAMTS3). Extensive research has investigated the developmental origins of blood endothelial cells (BECs) within the cardinal vein, which subsequently differentiate into lymphatic endothelial cells (LECs) [6,7,8,9,10,11]. The loss of Prox1 in paraxial mesoderm cells leads to the absence of dermal lymphatics in the lumbar region of embryos at embryonic day 15.5, accompanied by subcutaneous edema and blood-filled lymphatic capillaries in the cervical and thoracic dermis. Recent research using single-cell RNA analysis and lineage tracing suggests that LECs may directly originate from lymphangioblasts generated from the paraxial mesoderm. The sequence of differentiation of paraxial mesoderm cells into BECs in the anterior cardinal vein, followed by their development into LECs, as well as the potential differentiation of paraxial mesoderm-derived cells into specialized angioblasts before becoming LECs, remains the subject of ongoing controversy necessitating further investigation. Lymphatic valves form through the growth of lymphatic valve progenitor cells within the channel lumen, enclosing an extracellular matrix core. This intricate process is regulated by several transcription factors—PROX1, GATA2, FOXC2, and β-catenin, as documented in previous studies [12,13,14,15,16,17]. These factors, alongside KLF2 and KLF4, are upregulated by oscillatory shear stress [18]. VE-cadherin’s presence is crucial for activating mechanotransduction signaling pathways, leading to specific transcription factor upregulation. VE-cadherin’s binding partner, β-Catenin, is essential in determining valve regions during embryonic valve development [19]. A recent study identified FOXO1 as the primary suppressor of the lymphatic valve gene program [20]. The lack of FOXO1 leads to increased expression of valve-related genes such as Foxc2, Gata2, Klf4, and Klf2. FOXC2 plays a crucial role in lymphatic valve development and maintenance. When individuals have only one functional copy of the FOXC2 gene, they may develop lymphedema distichiasis. Notably, complete restoration of valve loss in Foxc2-heterozygous mice was achieved by deleting the Foxo1 gene, indicating potential translational applications of this protein. Understanding the development and coordination of lymphatic muscle cells (LMCs) in the pumping process of lymphatic precollector and collector, both in normal and pathological conditions, is a significant area of interest in lymphatic research (Figure 1). Muscle cell recruitment regulation is governed by PDGFB and angiopoietin-2, as well as semaphorin/neuropilin signaling through semaphorin 3A (SEMA3A) and neuropilin 1 (NRP1) [21,22,23,24]. Developing novel techniques for targeted removal of LMC genes would significantly advance future research efforts [24].

## 3. Lymphatics in Immune Regulation and Inflammation

Lymphatic system has a key role in adaptive immune responses and removal from tissues of triggers for harmful inflammatory reactions. This is due to the inclusion of lymph nodes along major lymphatic routes from tissues to the circulation. Through afferent lymphatics, lymph nodes receive antigens derived from the tissues. Lymph carries both lymphocytes and antigen-presenting cells (APCs), including monocytes and macrophages and dendritic cells, that process antigens for immune presentation in the lymph node [25,26]. Many antigens are foreign molecules. Their entry into the bloodstream relies on button-like junctions or mechanisms of endocytosis and transcytosis [27]. Lymphocytes, retained in the lymph node by loss of the receptor S1P1 for sphingosine 1 phosphate (S1P) from the cell surface [28], interact with the antigen-presenting cells through T cell receptors (TCRs) engagement with the cognate peptide major histocompatibility complex (MHC). In contrast to T cells, most APCs (that become infected) do not leave the lymph nodes, avoiding diffusion of infectious agent in the circulation and optimizing host defense. Even a low level of egress from lymph nodes can lead to negative outcomes [29]. Indeed, when these cells are activated, they upregulate molecules that initiate blood clotting, such as tissue factor [30], which initiates clotting in the extrinsic pathway of coagulation. Sepsis and disseminated intravascular coagulation are possible sequelae of outcomes that sequestration of myeloid APCs in lymph nodes prevents.

Inflammation is a mechanism of protection against pathogens [31] and is characterized by the expansion of blood and lymphatic networks. Angiogenesis exacerbates the inflammation, while lymphangiogenesis, by acting as clearance conduits, alleviating oedema, and decreasing the levels of pro-inflammatory mediators and immune cells, temper this condition [31]. Several molecular mechanisms involved in some inflammatory diseases of skin [32], bowel [33] and joints [34] have been related to lymphatic biology [35,36,37,38,39,40,41,42,43,44,45,46,47]. However, limited studies are available on developing agents able to promote lymphangiogesis and increase the potential of alleviation of inflammatory conditions. 

## 4. Kidney Lymphatic System 

**Figure 2:**
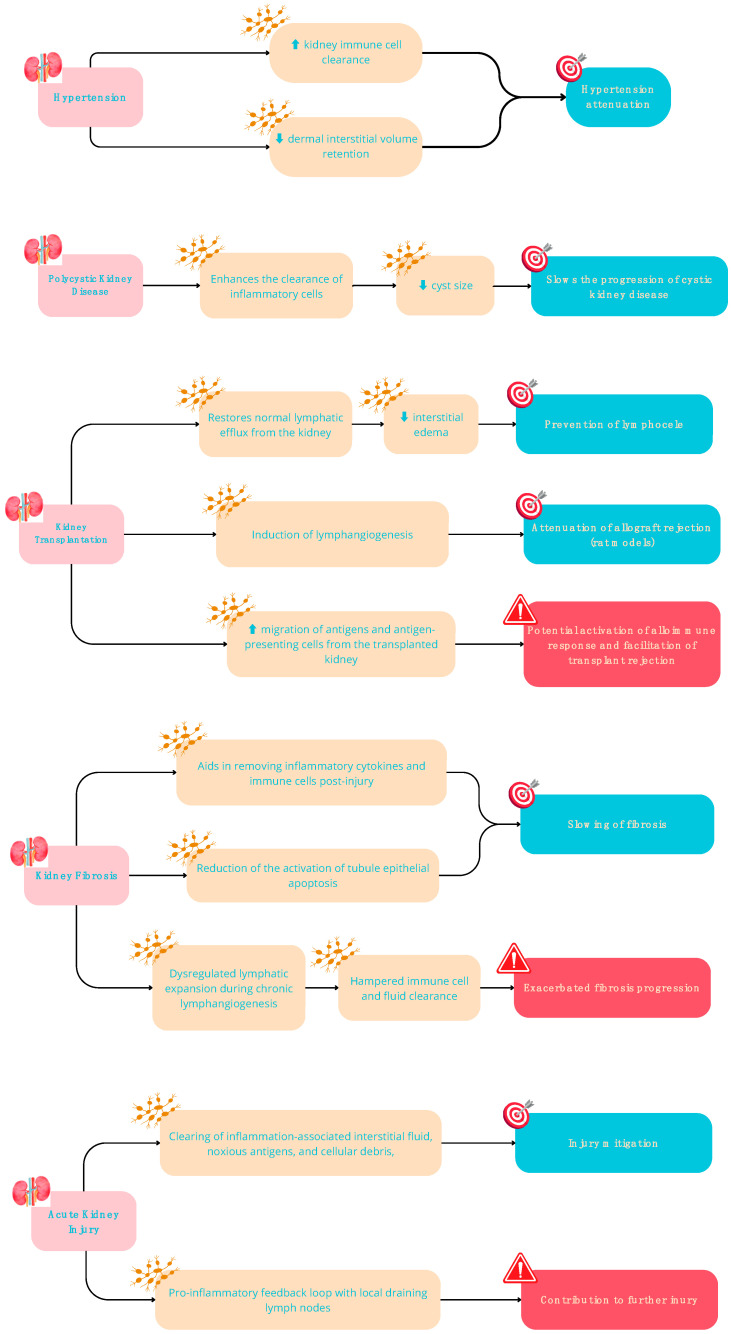
Potential Beneficial (in green) and Detrimental (in red) effects of lymphangiogenesis in kidney diseases.

The cortical lymphatic system in the kidney functions to manage interstitial fluid removal for fluid balance regulation. Simultaneously, it aids in the reabsorption of interstitial plasma elements such as proteins, electrolytes, cytokines, growth factors, and immune cells. Thus, kidney-derived lymph presents a blend of general and organ-specific components that mirror the kidney’s unique microenvironment. Analyses of lymphatic-to-plasma ratios of inulin [39], creatinine, labeled glucose, and electrolytes [48,49,50] upon exiting the kidney have defined the composition of kidney lymph. These studies revealed that kidney lymph comprises fluid, electrolytes, and small proteins from both capillary and tubular filtrates. Notably, their solute concentration resembles that of plasma [51]. Additionally, renin and angiotensin II, typically found in interstitial fluid, are also present in kidney lymph. Intriguingly, their concentrations in renal lymph might surpass those in venous plasma, hinting at a possible secretion of these substances into the kidney’s interstitial space [52,53,54]. However, the specific role of lymphatic transport in these molecules remains uncertain. The renal lymphatic system also harbors filtered antigens smaller than albumin and resident dendritic cells within the tubular compartment. An increase in pro-inflammatory cytokines and chemo-attractants in the lymphatic system occurs during kidney injury. All these proteins and cellular elements travel to nearby draining lymph nodes, contributing to maintaining peripheral immunological tolerance (Figure 2). 

## 5. Renal Lymphatic System Dysfunction

The role of the renal lymphatic system is pivotal across various facets of kidney function, including kidney development, immune responses, tissue fluid regulation, and maintaining fluid balance. Furthermore, it significantly influences the progression and persistence of renal ailments [53,54,55,56,57,58,59]. In adult kidneys, lymphatic capillaries are predominantly situated near glomeruli and tubules within the renal cortex, while classical lymphatic channels are not typically present in the renal medulla of healthy kidneys. However, renal lymphangiogenesis has been observed in various kidney diseases, such as polycystic kidney disease (PKD), transplant rejection, acute kidney injury, diabetic nephropathy, and IgA nephropathy [50]. Notably, there exists a correlation between renal lymphangiogenesis and the development of renal fibrosis in chronic kidney disease [60].

**Figure 3:**
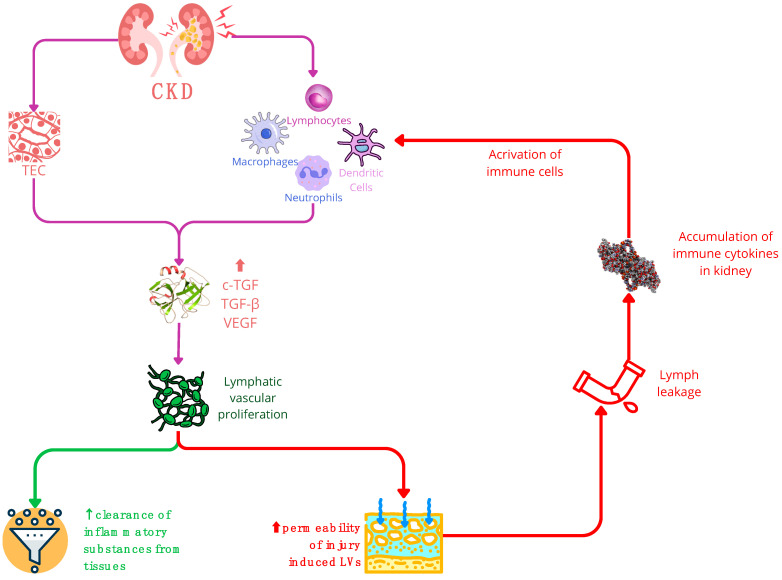
In chronic kidney disease (CKD), the stimulation of TGF triggers lymphatic vascular proliferation. Within CKD, TECs (tubular epithelial cells) and activated immune cells—such as macrophages, neutrophils, dendritic cells, and lymphocytes—serve as primary sources of growth factors like VEGF and TGF. Consequently, these factors promote lymphangiogenesis. The expansion of the lymphatic network in CKD yields a dual impact: a positive effect involves the increased LVs (lymphatic vessels) aiding in the clearance of inflammatory substances from tissues. Conversely, the negative influence arises from the hyperpermeability of injury-induced LVs, potentially causing lymph leakage and exacerbating the accumulation of immune cytokines in the kidneys. It is important to note that while increased LVs can aid in tissue clearance, their hyperpermeability may worsen the immune response in the kidney. Abbreviations: CKD—chronic kidney disease; cTGF—connective tissue growth factor; LEC—lymphatic endothelial cell; LN—lymph node; TEC—tubular epithelial cell; VEGF—vascular endothelial growth factor.

Despite a well-established association between renal lymphangiogenesis and multiple chronic kidney disorders, diverse observations on its outcomes have been noted. For instance, heightened renal function resulting from lymphangiogenesis can lead to conflicting consequences. While it aids in waste material and inflammatory cell clearance, benefiting the kidney, it might also exacerbate inflammatory responses by facilitating the transport of antigen-presenting cells to hilar lymph nodes, potentially harming the kidney [50]. Variations in disease outcomes might stem from changes in lymphatic function rather than solely the proliferation of nonfunctional lymphatics [60]. Notably, in certain scenarios, renal lymphangiogenesis has shown a protective effect in mouse models of polycystic kidney disease (PKD) and has the potential to regulate blood pressure [31,60,61]. For instance, administering recombinant VEGFC in mouse models of PKD augmented lymphangiogenesis, aiding in pericystic inflammatory cell removal and consequently reducing cystic disease [62]. Similarly, experiments involving the administration of VEGF-C in mice with normal genetic makeup, as well as investigations using animals with genetically modified tubules overexpressing Vascular Endothelial Growth Factor D (VEGF-D), showcased significant enhancements in natriuresis. These enhancements were accompanied by reductions in inflammation, kidney fibrosis, and hypertensive reactions [60]. Additional evidence supporting the idea that improved lymphatic function leads to decreased inflammatory reactions and preserved organ function is found in studies linking increased lymphatic density in renal transplant biopsies or the overexpression of VEGF-C in mouse models of kidney transplant to improved allograft function and transplant success [63] (Figure 3).

## 6. Lymphangiogenesis in Renal Inflammation Ad Fibrosis Development

The mature kidney contains a sparse network of blind-ended lymphatic capillaries adjacent to cortical tubules, arterioles and glomeruli. These capillaries drain out of the kidney through a hierarchical network of collecting lymphatics, which run in parallel with the larger interlobar and arcuate arteries and veins, and eventually drain from the hilar lymphatics [60]. In mouse models of chronic renal injury as well as in human chronic kidney disease, a process of expansion of lymphatics, i.e., lymphangiogenesis, occurs via the proliferation and sprouting of lymphatic endothelium [60,63]. In addition to lymphatic endothelial cells, the direct contribution by other cell types of nonvenous origin has been reported. Some studies suggest transdifferentiation of macrophages into lymphatic endothelial cells and their integration into lymphatic vessels, as an alternative mechanism of lymphatic expansion in chronic renal injury. The process is promoted by injured tubular epithelium, T and B lymphocytes, neutrophils, dendritic cells and activated fibroblasts that secrete growth factors (VEGF-C, VEGF-D, CTGF) and inflammatory mediators (lymphotoxin-a [LT-a], TNF-a, TGF-b) [60,63]. 

The primary driver of lymphatic growth is VEGF-C, both in development and in response to injury. Postdevelopmental lymphangiogenesis occurs in response to noxious stimuli, including interstitial fluid overload and inflammation and several kidney diseases, including immunoglobulin A nephropathy, diabetic nephropathy, polycystic kidney disease, and transplant rejection [60,63].

While intrarenal lymphangiogenesis is associated with renal interstitial inflammatory cell infiltration and fibrosis in patients with chronic kidney disease, the implications of lymphangiogenesis in these settings remain debated. 

Some evidence suggests that newly formed lymphatic vessels may be beneficial in clearing inflammation-associated accumulation of interstitial fluid, immune cell infiltrates, pro-inflammatory cytokines, and cellular debris, mitigating the progression of kidney disease [64]. In this scenario, the expansion of lymphatics could be teleologically interpreted as a means of tempering renal inflammation and, eventually, fibrotic remodeling. [65].

On the other hand, transport of APC via lymphatic vessels might putatively promote, as mentioned above, an inflammatory response. In this view, expanding renal lymphatics might play a critical role in promoting intrarenal inflammation and fibrosis following renal injury. 

A paradigmatic example of this controversial interpretation derives from transplantation. Lymphangiogenesis is typically associated with interstitial infiltration and fibrosis, but a retrospective review of kidney transplant protocol biopsies showed that higher kidney allograft lymphatic density was associated with better allograft function [60,63]. Moreover, in a mouse model of kidney transplantation with induced lymphangiogenesis within the allograft via forced overexpression of VEGF-C, transplant rejection was significantly reduced, and the lifespan of the recipient mice was improved [60].

Targeting lymphatic system could be a potential direction for new therapeutics for kidney diseases. However, a better understanding of the context-dependent consequences of kidney lymphangiogenesis is needed prior to their clinical use. 

## 7. Lymphangiogenesis Abnormalities and Genetic Disorders 

Genetic and epigenetic disorders affecting the development of the lymphatic vascular system can disrupt the formation and structural growth of lymphatic vessels. This disruption hampers the maintenance of the body’s fluid homeostasis, lipid reabsorption, and circulation of immune cells. Consequently, the impaired transport capacity of the lymphatic system leads to progressive fluid accumulation in the interstitial tissues and extracellular matrix throughout the body. The most common manifestation of this condition is primary lymphedema.

Primary lymphedema can manifest as a sporadic, systemic phenomenon or as a symptom associated with specific syndromes. It may be congenital or develop later in life, often resulting in chronic swelling and an increased risk of infections.

In recent years, the identification of numerous genetic variants, chromosomal abnormalities, and imprinting disorders observed in both systemic and syndromic lymphedema has led to the development of novel algorithms and classifications. These new approaches aim to correlate genotypes with phenotypes, enhancing our understanding and management of these conditions [66,67]. 

The primary phenotypes linking lymphatic abnormalities to kidney disorders include:

### 7.1. Hypotrichosis-Lymphedema-Telangiectasia-Renal Defect (HLTRS)

This extremely rare syndrome is associated with an autosomal dominant variant of the SOX18 gene on chromosome 20q13. Documented cases are characterized by alopecia, hydrocele from birth, late-onset lymphedema, and glomerulonephritis development. It is suggested that SOX18 gene mutations may disrupt the transcription of the SOX gene family, including SOX17 [68]. Experiments with double blockade of Sox17 and Sox18 in mice revealed reduced vascular abnormalities in the liver and kidney [69]. To date, only anecdotal reports are available. Among those, two families with children affected by a SOX18 mutation have been described: a living patient and his deceased brother from Canada, and a patient from Belgium. The two surviving patients, diagnosed with HLTS, were found through DNA analysis to share the same heterozygous C > A transversion in the SOX18 gene, leading to a premature truncation of the protein and the absence of the transactivation domain. Both individuals developed renal failure accompanied by severe hypertension in childhood, necessitating renal transplants for each [69]. These observations documented the renal failure linked to heterozygous SOX18 mutations. 

### 7.2. Lymphedema-Distichiasis Syndrome (LPHDST)

This syndrome is caused by an autosomal dominant variant of the FOX C2 gene at locus 16q24.1. It is marked by distichiasis (double rows of eyelashes) from birth and lower limb lymphedema appearing at prepubertal onset. Additional associated signs may include cardiac abnormalities, type II diabetes, and renal disorders like hydronephrosis, ectopic kidney, and renal agenesis. The 16q24.1–q24.2 microdeletion is linked with alveolar capillary dysplasia, congenital renal malformations, neurodevelopmental disorders, and congenital abnormalities [70,71]. 

### 7.3. Tuberous Sclerosis Complex (TSC) and Lymphedema

Variants in TSC1 and TSC2 genes, associated with Tuberous Sclerosis Complex, have been recently considered in the spectrum of molecular disorders related to primary lymphedema [72,73]. While the role of these genes in lymphatic system development remains unclear, rare cases of lymphedema in the lower limbs have been noted as symptoms of tuberous sclerosis [73]. TSC is an autosomal dominant multisystem disorder characterized by hamartomas in multiple organs, including the brain, skin, heart, kidneys, and lungs, with renal lesions typically presenting as angiomyolipomas, renal cysts, and renal-cell carcinomas.

## 8. Lymphangiogenesis and Peritoneal Ultrafiltration Failure

Peritoneal dialysis utilizes the patient’s peritoneal membrane to facilitate fluid and solute exchange. Waste materials diffuse across a network of capillaries lining the peritoneum, entering the dialysate solution introduced into the abdominal cavity. Concurrently, a hypertonic glucose solution and osmoles in the dialysate cause excess fluid to move from the peritoneal capillaries to the abdominal cavity, creating an osmotic gradient. This process, known as ultrafiltration, is counteracted by ongoing reabsorption through the peritoneal lymphatic capillaries, returning fluid to the vascular system. The cumulative ultrafiltration in peritoneal dialysis represents net fluid removal across the capillary walls, considering the reduction due to lymphatic reabsorption. Peritoneal ultrafiltration failure (UFF) is a consequential outcome characterized by increased peritoneal solute transport and reduced ultrafiltration capacity [74]. This factor significantly contributes to the discontinuation of peritoneal dialysis, which is strongly linked to heightened mortality rates. The emergence of UFF correlates with chronic peritoneal fibrosis, featuring submesothelial fibrosis and neo-angiogenesis. Poor ultrafiltration results from increased vascular surface area, greater permeability, and decreased channel-mediated water transport. However, although UFF is associated with neo-angiogenesis, the blood capillary density in peritoneal biopsies from individuals with UFF mirrors those without it. This suggests additional factors beyond blood capillary density influencing heightened peritoneal membrane fluid transport in UFF. Lymphangiogenesis, resulting in increased lymphatic fluid uptake from the peritoneal cavity, could play a significant role in UFF development. Comparing blood capillary density, lymphatic vessel quantity, and the rate of lymphatic absorption in the peritoneum reveals correlations with peritoneal dialysis duration and UFF occurrence. Human dialysate VEGF-C levels positively relate to peritoneal permeability. Inhibition of lymphangiogenesis using a soluble VEGFR-3 inhibitor enhanced compromised ultrafiltration in mice. Lymphangiogenesis in peritoneal uremic fluid is strongly associated with peritoneal fibrosis progression [74,75,76,77,78].

Chronic inflammatory diseases like peritonitis correlate with UFF, peritoneal fibrosis progression, and heightened lymphangiogenesis. Molecular processes governing peritoneal lymphangiogenesis, akin to those in other organs, involve TGFβ inducing lymphangiogenesis and fibrosis, with mesothelial cells and macrophages responding to TGFβ by secreting VEGF-C to stimulate lymphangiogenesis. CTGF, also released by activated peritoneal mesothelial cells due to TGFβ signaling, contributes to fibrosis and lymphangiogenesis. CTGF and VEGF-C exhibit a dynamic interaction; while CTGF limits VEGF-C binding to lymphatic endothelial cells (LECs), overall, CTGF promotes peritoneal lymphangiogenesis. Reducing CTGF inhibition lessens UFF and slows peritoneal fibrosis, presenting it as a potential therapeutic target. In contrast, inhibiting the VEGF-C-VEGFR-3 pathway improves compromised ultrafiltration without impacting peritoneal fibrosis progression. This indicates that fibrosis development, associated with CTGF, may involve mechanisms independent of VEGF-C. These mechanisms could involve CTGF’s role in angiogenesis and its direct profibrotic activity [73,74,75,76].

## 9. Therapeutical Considerations

The recent understating of the dynamic adaptability of the lymphatic vessels, along with their crucial role in controlling various vital bodily functions, including physiological processes, inflammation, and fibrosis, has sparked significant interest in the creation of treatments aimed at the lymphatic system. Currently, clinical trials are currently investigating various lymphatic-specific treatments for lymphedema and malignancies (as reviewed in [79,80,81,82,83]. 

While therapies targeting lymphatic aspects in kidney diseases are still in the early stages of development, a few show promising potential, as indicated by findings from preliminary animal studies. Main findings are summarised in Table 2 and Table 3.

Nevertheless, some considerations are still worth mentioning. Significant advancements have been achieved in understanding the regulation of the lymphatic vascular system, yet this knowledge still falls short when compared to our understanding of the blood vascular system. Further research is crucial to address key questions regarding both the fundamental mechanisms and, more importantly, the therapeutic implications of lymphatic regulation. A critical question remains: Can the pathological changes seen in chronic lymphedema, such as late-stage fibrosis, fat accumulation, and chronic inflammation, be reversed with prolymphangiogenic therapy? This inquiry is closely linked to determining the optimal duration for such therapy. For instance, initially, VEGF-C therapy may cause lymphatic vessel dysfunction, but developing a sufficiently dense network of lymphatic capillaries takes several days at minimum. Therefore, it is crucial to discontinue VEGF-C expression at an appropriate time to reduce edema and facilitate vessel remodeling [82,83]. This brief therapeutic window suggests that using VEGF-C as a recombinant protein, rather than through viral gene transfer vectors, might be preferable for more precise control over dosage and treatment duration. Future therapies will undergo rigorous safety evaluations. Patients with a high risk of dormant cancer must be precluded from treatment, although the use of specific prolymphangiogenic factors is expected to be well tolerated. Ideally, treatment effectiveness should be monitored using advanced imaging techniques, such as near-infrared, optical frequency-domain imaging, or real-time photoacoustic microscopy with an ultrasound array, which enable dynamic, quantitative, and qualitative imaging of the lymphatic vasculature. 

An intriguing strategy might combine prolymphangiogenic therapy with proangiogenic therapy, especially since the latter often leads to vascular leakage and subsequent edema, which could be alleviated by creating new lymphatic channels.

## 10. Conclusions

While there is a growing recognition of the importance of lymphatic vessels in various disease processes, substantial knowledge gaps persist regarding the functionality of the lymphatic system in both healthy and diseased states. There is an urgent need to deepen our understanding of how lymphatics function in safeguarding health and contributing to diseases in the kidney and other organs pertinent to nephrologists. The potential to develop innovative therapies that slow down fibrosis and cyst formation, along with improving the lifespan of the peritoneum for home dialysis, is highly promising. However, the effectiveness of these potential treatments is limited by our current understanding of how these signaling pathways operate uniquely in specific organs and under varying circumstances. The reactivation of organ-specific developmental signaling pathways commonly occurs in disease contexts. Extensive investigations into the origin and development of the kidney lymphatic system, employing advanced techniques such as single-cell RNA sequencing and lineage tracing, hold promise for pioneering novel therapeutic approaches in clinical settings.

## Figures and Tables

**Table 1:** Main genetic, molecular, and cellular pathways involved in developing lymphatic vessels.

Anatomic Site	Cellular Type	Involved Genes	External Factors	Cellular Markers	Notes
Lymphatic vessels	Embryonic venous blood endothelial cells (BECs)	Prox1, Sox18 and Coup-TFII activation			BECs derive from the anterior side of the cardinal vein. Prox1-knockout mice lack lymphatics and fail to thrive.
	Lymphatic endothelial cells (LECs)		ADAMTS3 and CCBE1 clivate VEGFC regulating its activity	α9 integrin VEGFR3 ^1^	LECs Derive from BECs α9 integrin and VEGFR3 facilitate migration towards mesenchymal cells guided by Vascular Endothelial Growth Factor C (VEGFC) gradient
				CCL21	CCL21 is expressed on the surface of LMCs after activation of VEGFR3. It has chemotactic properties for inflammatory cells, including dendritic cells
	Lymphatic muscle cells (LMCs)	SEMA3A NRP1	PDGFB angiopoietin-2		LMCs play a pivotal role in the pumping process of lymphatic pre-collector and collector PDGFB and angiopoietin-2 regulate their recruitment throughout SEMA3A NRP1
Lymphatic valves		PROX1, GATA2, FOXC2, KLF2 and KLF4 induce lymphatic valve formation		VE-cadherin and its binding partner β-catenin	Form through the growth of lymphatic valve progenitor cells within the channel lumen, enclosing an extracellular matrix core. The transcription factors genes are upregulated by oscillatory shear stress throughout VE-cadherin activity. Patients with only one functional copy of the FOXC2 gene may develop lymphedema distichiasis syndrome.
		FOXO1 suppress lymphatic valve formation			The lack of function of this gene leads to increased expression of valve-related genes such

SOX18: Sex-determining region Y box 18; COUP-TFII: Chicken ovalbumin upstream promoter transcription factor PROX1: Prospero homeobox 1; CCL21: C Chemokine 21; ADAMTS3: a disintegrin and metalloproteinase with thrombospondin motifs 3; CCBE1: collagen- and calcium-binding EGF domains 1; VEGFC: Vascular Endothelial Growth Factor Receptor 3; VEGFC: Vascular Endothelial Growth Factor C; SEMA3A: semaphorin 3A; NRP1: neuropilin 1; ^1^ VEGFR3 is expressed also by machrophages infiltrating the growing lymphatic vessels.

**Table 2:** Therapeutic targeting of the lymphatics.

Therapeutic Targeting	Comment	References
Blocking VEGF	In human studies, the effectiveness of Lymfactin^®^, an investigational gene therapy vector based on adenoviral type-5, encoding the expression of human VEGF-C, has been evaluated in both Phase I and Phase II clinical trials, in conjunction with VLNT (Vascularized Lymph Node Transfer) treatment. Notably, in a Phase I clinical trial involving 15 patients with upper limb lymphedema related to breast cancer (BCRL), conducted under trial identifier NCT02994771, no adverse events were reported during the 24-month follow-up period. In the higher-dose group, a significant 46% reduction in excess arm volume was observed 12 months after surgery, accompanied by a noteworthy improvement in quality-of-life scores. However, it is worth noting that the results of a Phase II double-blind, randomized, placebo-controlled, multicenter clinical trial (NCT03658967) were inconclusive, leading to the discontinuation of the drug’s development.	[84,85,86]
Podoplanin	Anti-podoplanin antibodies have been investigated in preclinical studies for the treatment of malignancies and thrombosis.	[87,88]
Connective tissue growth factor	FG-3019, which disrupts the activity of CTGF, is currently undergoing clinical trials in patients with pulmonary fibrosis.	[89,90]
Fibroblast growth factor 2 (FGF2)	In a rat tail model, a topical application of basic fibroblast growth factor (bFGF) to address secondary lymphedema was considered. This treatment led to elevated expression levels of VEGF-C/D, boosted lymphatic vessel density, reduced tail swelling, and enhanced lymphatic functionality.	[91]
Tacrolimus	The application of topical tacrolimus has been shown to inhibit CD4+ cell proliferation and differentiation by suppressing IL-2, as supported by several studies In mouse models of lymphedema, topical tacrolimus administration has demonstrated the ability to reduce inflammation, Th2 cytokines, fibroadipose tissue accumulation, swelling, and enhance lymphatic function. Notably, unlike oral administration, topical tacrolimus application did not lead to significant systemic absorption and did not produce significant systemic anti-inflammatory effects. In a recent open-label, single-arm, Phase II trial (NCT04541290), a 6-month treatment regimen with topical tacrolimus showed significant improvements in limb volumes, bioimpedance scores, and quality of life scores among eighteen women with breast cancer-related lymphedema (BCRL). However, the assessment of lymphatic function using ICG lymphography yielded inconclusive results.	[92,93]

**Table 3:** Therapeutic targeting of the lymphatics in renal diseases.

Therapeutic Targeting	Comment	References
Blocking VEGF	The use of a soluble VEGFR-3 inhibitor to block lymphangiogenesis significantly enhanced ultrafiltration in mice. A soluble VEGFR-3 fusion protein (sVEGFR3-FC), which captures and neutralizes VEGF-C and VEGF-D, likewise mitigated intrarenal lymphangiogenesis triggered by ureteral obstruction and ischemia-reperfusion, and it improved the progression of kidney fibrosis. In a diabetic kidney disease mouse model, SAR131675, a VEGFR inhibitor, hindered kidney lymphangiogenesis, glomerulosclerosis, and tubulointerstitial fibrosis. Activating the VEGF-C–VEGFR-3 pathway to stimulate lymphangiogenesis has been demonstrated to offer therapeutic advantages, such as diminishing kidney fibrosis and lessening the severity of cystic kidney disease in both mice and rats.	[41,46,65]
Podoplanin	In the context of kidney disease, the use of an anti-podoplanin antibody treatment was found to be protective against the development of kidney injury in a mouse model of nephrotoxic serum nephritis. In a particular study, the treatment disrupted the fibroblastic reticular cells’ ability to attract T cells, consequently preventing their activation within the kidney’s draining lymph nodes. This was associated with a significant reduction in the expansion of the lymphatic vasculature. Mice treated with the anti-podoplanin antibody showed improved kidney function, reduced infiltration of inflammatory macrophages, and a lower percentage of crescentic glomeruli compared to control mice who received a sham therapy.	[94]
Connective tissue growth factor	FG-3019 trial is ongoing. This trial opens up the possibility of targeting CTGF in the context of kidney fibrosis as well. In animal models of peritoneal fibrosis, FG-3019 has demonstrated its ability to prevent UFF (Uncontrolled Fibroblast Formation). However, it is yet to be determined to what extent the reduction of UFF in this context is attributed to the anti-lymphangiogenic properties of FG-3019 versus its impacts on angiogenesis and fibroblast activities.	[89,90]

## Data Availability

Not applicable due to the review nature of this manuscript.

## References

[1] Wigle J.T., Oliver G. (1999). Prox1 function is required for the development of the murine lymphatic system. Cell.

[2] Oliver G. (2004). Lymphatic vasculature development. Nat. Rev. Immunol..

[3] Srinivasan R.S., Dillard M.E., Lagutin O.V., Lin F.-J., Tsai S., Tsai M.-J., Samokhvalov I.M., Oliver G. (2007). Lineage tracing demonstrates the venous origin of the mammalian lymphatic vasculature. Genes Dev..

[4] Yaniv K., Isogai S., Castranova D., Dye L., Hitomi J., Weinstein B.M. (2006). Live imaging of lymphatic development in the zebrafish. Nat. Med..

[5] Wigle J.T., Harvey N., Detmar M., Lagutina I., Grosveld G., Gunn M.D., Jackson D.G., Oliver G. (2002). An essential role for Prox1 in the induction of the lymphatic endothelial cell phenotype. EMBO J..

[6] Mishima K., Watabe T., Saito A., Yoshimatsu Y., Imaizumi N., Masui S., Hirashima M., Morisada T., Oike Y., Araie M. (2007). Prox1 induces lymphatic endothelial differentiation via integrin alpha9 and other signaling cascades. Mol. Biol. Cell.

[7] Mehrara B.J., Radtke A.J., Randolph G.J., Wachter B.T., Greenwel P., Rovira I.I., Galis Z.S., Muratoglu S.C. (2023). The emerging importance of lymphatics in health and disease: An NIH workshop report. J. Clin. Investig..

[8] Klotz L., Norman S., Vieira J.M., Masters M., Rohling M., Dubé K.N., Bollini S., Matsuzaki F., Carr C.A., Riley P.R. (2015). Cardiac lymphatics are heterogeneous in origin and respond to injury. Nature.

[9] Martinez-Corral I., Ulvmar M.H., Stanczuk L., Tatin F., Kizhatil K., John S.W., Alitalo K., Ortega S., Makinen T. (2015). Nonvenous origin of dermal lymphatic vasculature. Circ. Res..

[10] Pichol-Thievend C., Betterman K.L., Liu X., Ma W., Skoczylas R., Lesieur E., Bos F.L., Schulte D., Schulte-Merker S., Hogan B.M. (2018). A blood capillary plexus-derived population of progenitor cells contributes to genesis of the dermal lymphatic vasculature during embryonic development. Development.

[11] Stone O.A., Stainier D.Y.R. (2019). Paraxial mesoderm is the major source of lymphatic endothelium. Dev. Cell.

[12] Sabine A., Petrova T.V. (2013). Interplay of mechanotransduction, FOXC2, connexins, and calvineurin signaling in lymphatic valve formation. Dev. Asp. Lymphat. Vasc. Syst..

[13] Kiefer F., Schulte-Merker S. (2014). Developmental Aspects of the Lymphatic Vascular System.

[14] Kazenwadel J., Secker G.A., Liu Y.J., Rosenfeld J.A., Wildin R.S., Cuellar-Rodriguez J., Hsu A.P., Dyack S., Fernandez C.V., Chong C.-E. (2012). Loss-of-function germline GATA2 mutations in patients with MDS/AML or MonoMAC syndrome and primary lymphedema reveal a key role for GATA2 in the lymphatic vasculature. Blood.

[15] Cha B., Geng X., Mahamud R., Zhang J.Y., Chen L., Kim W., Jho E.-H., Kim Y., Choi D., Dixon J.B. (2018). Complementary Wnt sources regulate lymphatic vascular development via PROX1-dependent Wnt/β-catenin signaling. Cell Rep..

[16] Norrmén C., Ivanov K.I., Cheng J., Zangger N., Delorenzi M., Jaquet M., Miura N., Puolakkainen P., Horsley V., Hu J. (2009). FOXC2 controls formation and maturation of lymphatic collecting vessels through cooperation with NFATc1. J. Cell Biol..

[17] Bazigou E., Lyons O.T., Smith A., Venn G.E., Cope C., Brown N.A., Makinen T. (2011). Genes regulating lymphangiogenesis control venous valve formation and maintenance in mice. J. Clin. Investig..

[18] Choi D., Park E., Jung E., Seong Y.J., Yoo J., Lee E., Hong M., Lee S., Ishida H., Burford J. (2017). Laminar flow downregulates Notch activity to promote lymphatic sprouting. J. Clin. Investig..

[19] Yang Y., Cha B., Motawe Z.Y., Srinivasan R.S., Scallan J.P. (2019). VE-cadherin is required for lymphatic valve formation and maintenance. Cell Rep..

[20] Scallan J.P., Knauer L.A., Hou H., Castorena-Gonzalez J.A., Davis M.J., Yang Y. (2021). Foxo1 deletion promotes the growth of new lymphatic valves. J. Clin. Investig..

[21] Bell R., Brice G., Child A., Murday V., Mansour S., Sandy C., Collin J., Brady A., Callen D., Burnand K. (2001). Analysis of lymphoedema-distichiasis families forFOXC2 mutations reveals small insertions and deletions throughout the gene. Hum. Genet..

[22] Kriederman B.M., Myloyde T.L., Witte M.H., Dagenais S.L., Witte C.L., Rennels M., Bernas M.J., Lynch M.T., Erickson R.P., Caulder M.S. (2003). FOXC2 haploinsufficient mice are a model for human autosomal dominant lymphedema-distichiasis syndrome. Hum. Mol. Genet..

[23] Wang Y., Jin Y., Mäe M.A., Zhang Y., Ortsäter H., Betsholtz C., Mäkinen T., Jakobsson L. (2017). Smooth muscle cell recruitment to lymphatic vessels requires PDGFB and impacts vessel size but not identity. Development.

[24] Jurisic G., Maby-El Hajjami H., Karaman S., Ochsenbein A.M., Alitalo A., Siddiqui S.S., Pereira C.O., Petrova T.V., Detmar M. (2012). An unexpected role of semaphorin3a-neuropilin-1 signaling in lymphatic vessel maturation and valve formation. Circ. Res..

[25] Davis M.J., Kim H.J., Li M., Zawieja S.D. (2023). A vascular smooth muscle-specific integrin-α8 Cre mouse for lymphatic contraction studies that allows male-female comparisons and avoids visceral myopathy. Front. Physiol..

[26] Randolph G.J., Ivanov S., Zinselmeyer B.H., Scallan J.P. (2017). The Lymphatic System: Integral Roles in Immunity. Annu. Rev. Immunol..

[27] Kähäri L., Fair-Mäkelä R., Auvinen K., Rantakari P., Jalkanen S., Ivaska J., Salmi M. (2019). Transcytosis route mediates rapid delivery of intact antibodies to draining lymph nodes. J. Clin. Investig..

[28] Cyster J.G., Schwab S.R. (2012). Sphingosine-1-phosphate and lymphocyte egress from lymphoid organs. Annu. Rev. Immunol..

[29] St John A.L., Ang W.X.G., Huang M.N., Kunder C.A., Chan E.W., Gunn M.D., Abraham S.N. (2014). S1P-Dependent trafficking of intracellular yersinia pestis through lymph nodes establishes Buboes and systemic infection. Immunity.

[30] Grover S.P., Mackman N. (2018). Tissue Factor: An Essential Mediator of Hemostasis and Trigger of Thrombosis. Arter. Thromb. Vasc. Biol..

[31] Schwager S., Detmar M. (2019). Inflammation and Lymphatic Function. Front. Immunol..

[32] Lane R.S., Femel J., Breazeale A.P., Loo C.P., Thibault G., Kaempf A., Mori M., Tsujikawa T., Chang Y.H., Lund A.W. (2018). IFNγ-activated dermal lymphatic vessels inhibit cytotoxic T cells in melanoma and inflamed skin. J. Exp. Med..

[33] Ge Y., Li Y., Gong J., Zhu W. (2018). Mesenteric organ lymphatics and inflammatory bowel disease. Ann. Anat.-Anat. Anz..

[34] Bouta E.M., Bell R.D., Rahimi H., Xing L., Wood R.W., Bingham C.O., Ritchlin C.T., Schwarz E.M. (2018). Targeting lymphatic function as a novel therapeutic intervention for rheumatoid arthritis. Nat. Rev. Rheumatol..

[35] Aldrich M.B., Velasquez F.C., Kwon S., Azhdarinia A., Pinkston K., Harvey B.R., Chan W., Rasmussen J.C., Ross R.F., Fife C.E. (2017). Lymphatic delivery of etanercept via nanotopography improves response to collagen-induced arthritis. Arthritis Res. Ther..

[36] Imai H., Yoshida S., Uchiki T., Sasaki A., Nagamatsu S., Koshima I. (2021). Successful Treatment of Rheumatoid Lymphedema with Lymphatic Venous Anastomosis. Plast. Reconstr. Surg. Glob. Open.

[37] Tily H.I., Perl A. (2009). Lymphoedema: A paradoxical effect of tumour necrosis factor inhibitors—Case report and review of literature. BMJ Case Rep..

[38] Chen Y., Rehal S., Roizes S., Zhu H., Cole W.C., von der Weid P. (2017). The pro-inflammatory cytokine TNF-α inhibit lymphatic pumping via activation of the NF-κB-iNOS signaling pathway. Microcirculation.

[39] Machnik A., Neuhofer W., Jantsch J., Dahlmann A., Tammela T., Machura K., Park J.K., Beck F.X., Müller D.N., Derer W. (2009). Macrophages regulate salt-dependent volume and blood pressure by a vascular endothelial growth factor-C-dependent buffering mechanism. Nat. Med..

[40] Sakamoto I., Ito Y., Mizuno M., Suzuki Y., Sawai A., Tanaka A., Maruyama S., Takei Y., Yuzawa Y., Matsuo S. (2009). Lymphatic vessels develop during tubulointerstitial fibrosis. Kidney Int..

[41] Hasegawa S., Nakano T., Torisu K., Tsuchimoto A., Eriguchi M., Haruyama N., Masutani K., Tsuruya K., Kitazono T. (2017). Vascular endothelial growth factor-C ameliorates renal interstitial fibrosis through lymphangiogenesis in mouse unilateral ureteral obstruction. Lab. Investig..

[42] Huang J.L., Woolf A.S., Kolatsi-Joannou M., Baluk P., Sandford R.N., Peters D.J., McDonald D.M., Price K.L., Winyard P.J., Long D.A. (2016). Vascular Endothelial Growth Factor C for Polycystic Kidney Diseases. J. Am. Soc. Nephrol..

[43] Zarjou A., Black L.M., Bolisetty S., Traylor A.M., Bowhay S.A., Zhang M.Z., Harris R.C., Agarwal A. (2019). Dynamic signature of lymphangiogenesis during acute kidney injury and chronic kidney disease. Lab. Investig..

[44] Abouelkheir G.R., Upchurch B.D., Rutkowski J.M. (2017). Lymphangiogenesis: Fuel, smoke, or extinguisher of inflammation’s fire?. Exp. Biol. Med..

[45] Choi S.Y., Lim S.W., Salimi S., Yoo E.J., Lee-Kwon W., Lee H.H., Lee J.H., Mitchell B.D., Sanada S., Parsa A. (2018). Tonicity-Responsive Enhancer-Binding Protein Mediates Hyperglycemia-Induced Inflammation and Vascular and Renal Injury. J. Am. Soc. Nephrol..

[46] Wang S.D., Song J.H., Kim Y., Lim J.H., Kim M.Y., Kim E.N., Hong Y.A., Chung S., Choi B.S., Kim Y.S. (2019). Inhibition of lymphatic proliferation by the selective VEGFR-3 inhibitor SAR131675 ameliorates diabetic nephropathy in db/db mice. Cell Death Dis..

[47] Rahimi H., Bell R., Bouta E.M., Wood R.W., Xing L., Ritchlin C.T., Schwarz E.M. (2016). Lymphatic imaging to assess rheumatoid flare: Mechanistic insights and biomarker potential. Arthritis Res. Ther..

[48] Keyl M.J., Scott J.B., Dabney J.M., Haddy F.J., Harvey R.B., Bell R.D., Ginn H.E. (1965). Composition of canine renal hilar lymph. Am. J. Physiol..

[49] Cockett A.T., Roberts A.P., Moore R.S. (1969). Renal lymphatic transport of fluid and solutes. Investig. Urol..

[50] Cook V.L., Reese A.H., Wilson P.D., Pinter G.G. (1982). Access of reabsorbed glucose to renal lymph. Experientia.

[51] Bell R.D. (1984). Renal lymph flow and composition during acetazolamide and furosemide diuresis. Lymphology.

[52] McIntosh G.H., Morris B. (1971). The lymphatics of the kidney and the formation of renal lymph. J. Physiol..

[53] Wilcox C.S., Peart W.S. (1987). Release of renin and angiotensin II into plasma and lymph during hyperchloremia. Am. J. Physiol..

[54] Bertoldi G., Caputo I., Calò L., Rossitto G. (2023). Lymphatic vessels and the renin-angiotensin-system. Am. J. Physiol. Heart Circ. Physiol..

[55] Niiro G.K., Jarosz H.M., O’Morchoe P.J., O’Morchoe C.C. (1986). The renal cortical lymphatic system in the rat, hamster, and rabbit. Am. J. Anat..

[56] O’Morchoe C.C., O’Morchoe P.J. (1988). The renal lymphatic system: A brief review. Contrib. Nephrol..

[57] Russell P.S., Itkin M., Windsor J.A., Phillips A.R.J. (2023). Kidney Lymphatics. Compr. Physiol..

[58] Dorraji S.E., Kanapathippillai P., Hovd A.K., Stenersrød M.R., Horvei K.D., Ursvik A., Figenschau S.L., Thiyagarajan D., Fenton C.G., Pedersen H.L. (2020). Kidney Tertiary Lymphoid Structures in Lupus Nephritis Develop into Large Interconnected Networks and Resemble Lymph Nodes in Gene Signature. Am. J. Pathol..

[59] Jafree D.J., Moulding D., Kolatsi-Joannou M., Perretta Tejedor N., Price K.L., Milmoe N.J., Walsh C.L., Correra R.M., Winyard P.J., Harris P.C. (2019). Spatiotemporal dynamics and heterogeneity of renal lymphatics in mammalian development and cystic kidney disease. eLife.

[60] Albertine K.H., O’Morchoe C.C. (1979). Distribution and density of the canine renal cortical lymphatic system. Kidney Int..

[61] Donnan M.D., Kenig-Kozlovsky Y., Quaggin S.E. (2021). The lymphatics in kidney health and disease. Nat. Rev. Nephrol..

[62] Liu J., Yu C. (2022). Lymphangiogenesis and Lymphatic Barrier Dysfunction in Renal Fibrosis. Int. J. Mol. Sci..

[63] Karihaloo A., Koraishy F., Huen S.C., Lee Y., Merrick D., Caplan M.J., Somlo S., Cantley L.G. (2011). Macrophages promote cyst growth in polycystic kidney disease. J. Am. Soc. Nephrol..

[64] Pedersen M.S., Müller M., Rülicke T., Leitner N., Kain R., Regele H., Wang S., Gröne H.J., Rong S., Haller H. (2020). Lymphangiogenesis in a mouse model of renal transplant rejection extends life span of the recipients. Kidney Int..

[65] Pei G., Yao Y., Yang Q., Wang M., Wang Y., Wu J., Wang P., Li Y., Zhu F., Yang J. (2019). Lymphangiogenesis in kidney and lymph node mediates renal inflammation and fibrosis. Sci. Adv..

[66] Jafree D.J., Long D.A. (2020). Beyond a Passive Conduit: Implications of Lymphatic Biology for Kidney Diseases. J. Am. Soc. Nephrol..

[67] Gordon K., Varney R., Keeley V., Riches K., Jeffery S., Van Zanten M., Mortimer P., Ostergaard P., Mansour S. (2020). Update and audit of the St George’s classification algorithm of primary lymphatic anomalies: A clinical and molecular approach to diagnosis. Med. Genet..

[68] Martin-Almedina S., Mortimer P.S., Ostergaard P. (2021). Development and physiological functions of the lymphatic system: Insights from human genetic studies of primary Lymphedema. Physiol. Rev..

[69] Moalem S., Brouillard P., Kuypers D., Legius E., Harvey E., Taylor G., Francois M., Vikkula M., Chitayat D. (2015). Hypotrichosis-lymphedema-telangiectasia-renal defect associated with a truncating mutation in the SOX18 gene. Clin. Genet..

[70] Matsui T., Kanai-Azuma M., Hara K., Matoba S., Hiramatsu R., Kawakami H., Kurohmaru M., Koopman P., Kanai Y. (2006). Redundant roles of Sox17 and Sox18 in postnatal angiogenesis in mice. J. Cell Sci..

[71] Michelson M., Lidzbarsky G., Nishri D., Israel-Elgali I., Berger R., Gafner M., Shomron N., Lev D., Goldberg Y. (2022). Microdeletion of 16q24.1–q24.2—A unique etiology of Lymphedema–Distichiasis syndrome and neurodevelopmental disorder. Am. J. Med. Genet..

[72] Jones G.E. (2017). Renal anomalies and lymphedema distichiasis syndrome. A rare association?. Am. J. Med. Genet..

[73] Brouillard P., Witte M.H., Erickson R.P., Damstra R.J., Becker C., Quéré I., Vikkula M. (2021). Primary lymphoedema. Nat. Rev. Dis. Primers..

[74] Klinner J., Krüger M., Brunet T., Makowski C., Riedhammer K.M., Mollweide A., Wagner M., Hoefele J. (2020). Congenital lymphedema as a rare and first symptom of tuberous sclerosis complex. Gene.

[75] Li J., Liu Y., Liu J. (2023). A review of research progress on mechanisms of peritoneal fibrosis related to peritoneal dialysis. Front. Physiol..

[76] Kinashi H., Toda N., Sun T., Nguyen T.Q., Suzuki Y., Katsuno T., Yokoi H., Aten J., Mizuno M., Maruyama S. (2019). Connective tissue growth factor is correlated with peritoneal lymphangiogenesis. Sci. Rep..

[77] Vlahu C.A., de Graaff M., Aten J., Struijk D.G., Krediet R.T. (2015). Lymphangiogenesis and LymphaticAbsorption Are Related and Increased in Chronic Kidney Failure, Independent of Exposure to Dialysis Solutions. Adv. Perit. Dial..

[78] Terabayashi T., Ito Y., Mizuno M., Suzuki Y., Kinashi H., Sakata F., Tomita T., Iguchi D., Tawada M., Nishio R. (2015). Vascular endothelial growth factor receptor-3 is a novel target to improve net ultrafiltration in methylglyoxal-induced peritoneal injury. Lab. Investig..

[79] Kinashi H., Ito Y., Mizuno M., Suzuki Y., Terabayashi T., Nagura F., Hattori R., Matsukawa Y., Mizuno T., Noda Y. (2013). TGF-β1 promotes lymphangiogenesis during peritoneal fibrosis. J. Am. Soc. Nephrol..

[80] Drobot D., Leitner Shemy O., Zeltzer A.A. (2024). Biomaterials in the clinical treatment of lymphedema a systematic review. J. Vasc. Surg. Venous. Lymphat. Disord..

[81] Brown S., Campbell A.C., Kuonqui K., Sarker A., Park H.J., Shin J., Kataru R.P., Coriddi M., Dayan J.H., Mehrara B.J. (2023). The Future of Lymphedema: Potential Therapeutic Targets for Treatment. Curr. Breast Cancer Rep..

[82] Senger J.B., Kadle R.L., Skoracki R.J. (2023). Current Concepts in the Management of Primary Lymphedema. Medicina.

[83] Norrmén C., Tammela T., Petrova T.V., Alitalo K. (2011). Biological basis of therapeutic lymphangiogenesis. Circulation.

[84] Hartiala P., Suominen S., Suominen E., Kaartinen I., Kiiski J., Viitanen T., Alitalo K., Saarikko A.M. (2020). Phase 1 Lymfactin^Ⓡ^ Study: Short-term Safety of Combined Adenoviral VEGF-C and Lymph Node Transfer Treatment for Upper Extremity Lymphedema. J. Plast. Reconstr. Aesthet. Surg..

[85] Fane M.E., Ecker B.L., Kaur A., Marino G.E., Alicea G.M., Douglass S.M., Chhabra Y., Webster M.R., Marshall A., Colling R. (2020). sFRP2 Supersedes VEGF as an Age-related Driver of Angiogenesis in Melanoma, Affecting Response to Anti-VEGF Therapy in Older Patients. Clin. Cancer Res..

[86] Leppäpuska I.M., Hartiala P., Suominen S., Suominen E., Kaartinen I., Mäki M., Seppänen M., Kiiski J., Viitanen T., Lahdenperä O. (2022). Phase 1 Lymfactin^®^ Study: 24-month Efficacy and Safety Results of Combined Adenoviral VEGF-C and Lymph Node Transfer Treatment for Upper Extremity Lymphedema. J. Plast. Reconstr. Aesthet. Surg..

[87] Payne H., Ponomaryov T., Watson S.P., Brill A. (2017). Mice with a deficiency in CLEC-2 are protected against deep vein thrombosis. Blood.

[88] Krishnan H., Rayes J., Miyashita T., Ishii G., Retzbach E.P., Sheehan S.A., Takemoto A., Chang Y.W., Yoneda K., Asai J. (2018). Podoplanin: An emerging cancer biomarker and therapeutic target. Cancer Sci..

[89] Sakai N., Nakamura M., Lipson K.E., Miyake T., Kamikawa Y., Sagara A., Shinozaki Y., Kitajima S., Toyama T., Hara A. (2017). Inhibition of CTGF ameliorates peritoneal fibrosis through suppression of fibroblast and myofibroblast accumulation and angiogenesis. Sci. Rep..

[90] Raghu G., Scholand M.B., de Andrade J., Lancaster L., Mageto Y., Goldin J., Brown K.K., Flaherty K.R., Wencel M., Wanger J. (2016). FG-3019 anti-connective tissue growth factor monoclonal antibody: Results of an open-label clinical trial in idiopathic pulmonary fibrosis. Eur. Respir. J..

[91] Onishi T., Nishizuka T., Kurahashi T., Arai T., Iwatsuki K., Yamamoto M., Hirata H. (2014). Topical bFGF Improves Secondary Lymphedema through Lymphangiogenesis in a Rat Tail Model. Plast. Reconstr. Surg. Glob. Open.

[92] Gardenier J.C., Kataru R.P., Hespe G.E., Savetsky I.L., Torrisi J.S., Nores G.D.G., Jowhar D.K., Nitti M.D., Schofield R.C., Carlow D.C. (2017). Topical tacrolimus for the treatment of secondary lymphedema. Nat. Commun..

[93] Gulmark Hansen F.C., Jørgensen M.G., Sørensen J.A. (2023). Treatment of breast cancer-related lymphedema with topical tacrolimus: A prospective, open-label, single-arm, phase II pilot trial. J. Breast Cancer.

[94] Kasinath V., Yilmam O.A., Uehara M., Jiang L., Ordikhani F., Li X., Salant D.J. (2019). AbdivR Activation of fibroblastic reticular cells in kidney lymph node duringvcrescentic glomerulonephritis. Kidney Int..

